# Comparative label-free lipidomic analysis of *Mycobacterium tuberculosis* during dormancy and reactivation

**DOI:** 10.1038/s41598-019-40051-5

**Published:** 2019-03-06

**Authors:** Sajith Raghunandanan, Leny Jose, Vipin Gopinath, Ramakrishnan Ajay Kumar

**Affiliations:** Mycobacterium Research Laboratory, Rajiv Gandhi Centre for Biotechnology, Thycaud P.O., Thiruvananthapuram, 695014 India

## Abstract

*Mycobacterium tuberculosis* employs several strategies to combat and adapt to adverse conditions encountered inside the host. The non-replicative dormant state of the bacterium is linked to drug resistance and slower response to anti-tubercular therapy. It is known that alterations in lipid content allow dormant bacteria to acclimatize to cellular stress. Employing comparative lipidomic analysis we profiled the changes in lipid metabolism in *M. tuberculosis* using a modified Wayne’s model of hypoxia-induced dormancy. Further we subjected the dormant bacteria to resuscitation, and analyzed their lipidomes until the lipid profile was similar to that of normoxially grown bacteria. An enhanced degradation of cell wall-associated and cytoplasmic lipids during dormancy, and their gradual restoration during reactivation, were clearly evident. This study throws light on distinct lipid metabolic patterns that *M*. *tuberculosis* undergoes to maintain its cellular energetics during dormancy and reactivation.

## Introduction

Tuberculosis (TB) is a dreadful disease caused by *Mycobacterium tuberculosis* (MTB). The death toll caused by MTB is on the rise and resulted in 1.6 million deaths in 2017^1^. The success of MTB as an intracellular pathogen has been attributed mainly to its ability to reprogram host cell functions to prevent its own destruction and subsequently to form granulomas where the bacteria can remain in a quiescent state^2,3^. Once inside the granuloma MTB ceases to divide, shuts down its central metabolism and enters a phase of dormancy rendering itself extremely resistant to host defense mechanisms and drug treatment^4–6^ which is one of the key reasons for the failure of current anti-TB drugs to eradicate TB. The phenomenon of dormancy in MTB has been studied extensively using genomic^7–11^ and proteomic^12–14^ approaches. Galagan *et al*. (2013) carried out a system-wide profiling of the transcriptome, proteome and metabolome of MTB during hypoxia and re-aeration, and observed significant changes in the lipid content^15^. A previous study from our laboratory found variations in the relative quantities of proteins involved in lipid metabolism during these stages^16^. Genes involved in fatty acid metabolism constitute a significant share (approximately 250 genes) of the MTB genome^17^. Fatty acids such as mycolic acids are the key components of the cell wall and are also immunomodulatory molecules^18^. Sartain *et al*. (2011) created a database (MTB LipidDB) that contains 2512 lipid entities^19^ of MTB whereas Layre *et al*. (2011) developed two other databases, MycoMap and MycoMass, using LC-MS-based approaches which contain more than 5000 molecular species of lipids of MTB with high mass accuracy and precision^20^. Both these databases follow the LIPID MAPS organizational tree (www.lipidmaps.org) and use the MTB lipid subclass level 4^21^. Although data on the lipidome of normoxially grown MTB are available, there is very little information about the differences in the total lipid profile during the transition from normoxial growth to dormancy and from dormancy to reactivated state.

In the present study we used a modified Wayne’s model of self-generated hypoxia to compare the lipidomes of MTB H37Rv during normoxia, dormancy and reactivation. Employing a high-throughput UPLC-MS analysis we observed significant alterations in the lipid profiles across the different stages. To substantiate the results we isolated mycolic acids from all these stages and verified their status using MALDI-TOF/MS. The results suggest that lipids are rapidly depleted during the establishment of dormancy and the phenomenon is reversed as the bacteria are re-aerated. The subsets of lipids that are present in each stage provide critical insights into the lipid biosynthetic as well as degradative pathways involved in mycobacterial persistence and the cellular machinery that controls them.

## Results

### Establishment of dormancy and reactivation *in vitro*

**Figure 1:**
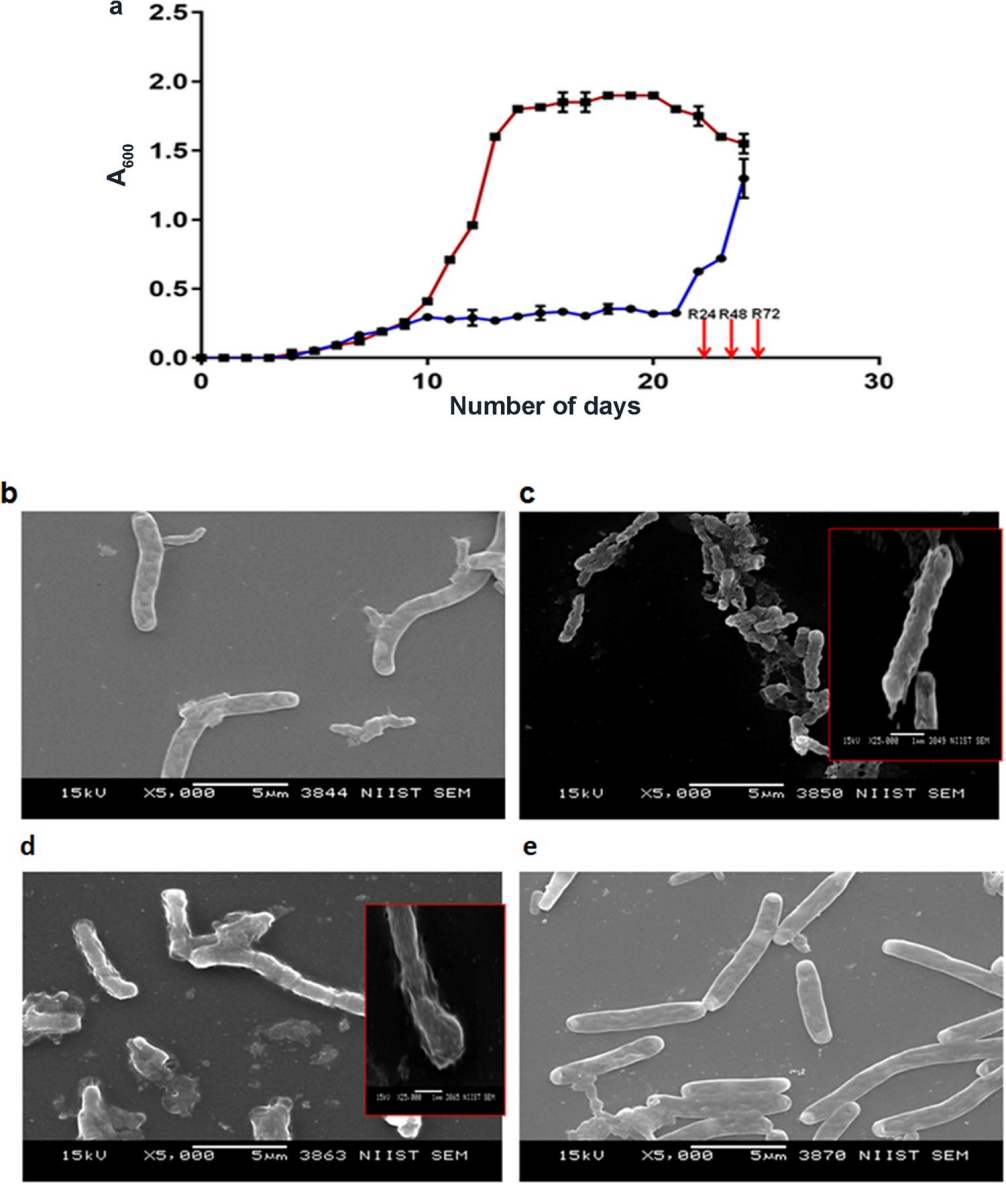
*In vitro* dormancy and reactivation of MTB. Growth of MTB H37Rv subjected to hypoxia and re-aeration (**a**). Brown line indicates OD (A600 nm) of normoxially grown MTB H37Rv. Purple line indicates MTB grown under hypoxic condition and reactivation. Arrows indicate time points of re-aeration. The error bars indicate standard deviations of the mean optical densities (n = 2). Morphology of MTB subjected to dormancy and re-aeration (**b–e**). Scanning electron microscopic (SEM) images showing MTB from normoxia (log phase) (**b**), dormancy (**c**), R24 (**d**) and R144 (**e**) stages; Magnification-5000X for main images and 10000X for inlay images. Scale bars are 5 and 1 µm for 5000X and 10000X, respectively.

In the present study we adopted a modified Wayne’s model for inducing dormancy and reactivation in MTB^16^. The self-generated hypoxia was monitored using methylene blue. The initial blue color gradually became green on day eight indicating establishment of a microaerophilic state and finally became colorless indicating attainment of strict anaerobic condition on day 14, and was maintained up to 21 days. Compared to aerobically grown MTB the growth pattern of bacteria under hypoxia was strikingly distinct showing gradual and sustained growth arrest and rapidly responded to re-aeration (Fig. 1a). To determine if MTB subjected to dormancy and reactivation undergoes any morphological changes, we visualized the bacteria under scanning electron microscope (SEM). Normoxially grown MTB showed typical morphology with a length of 5–7 µm (Fig. 1b). A drastic decrease in size was observed in dormant bacteria (1–2 µm) (Fig. 1c). Cavitation of the cell wall imparted a fragile appearance to the dormant cells. Upon re-aeration the bacterial morphology started to revert at 24 h (Fig. 1d). At 144 h post re-aeration the bacterial length as well as the morphology were comparable to those of normoxially grown MTB (Fig. 1e).

### Label-free comparative lipidomics and meta-analysis

To compare the lipid profile of the normoxially grown MTB with that of dormant and reactivated bacteria, we isolated total lipids from MTB during all these conditions and analyzed them by label-free lipidomics analysis using high resolution UPLC-MS. All the raw files, assays, study design and spectral files are available on MetaboLights server (http://www.ebi.ac.uk/metabolights/MTBLS304).

**Figure 2:**
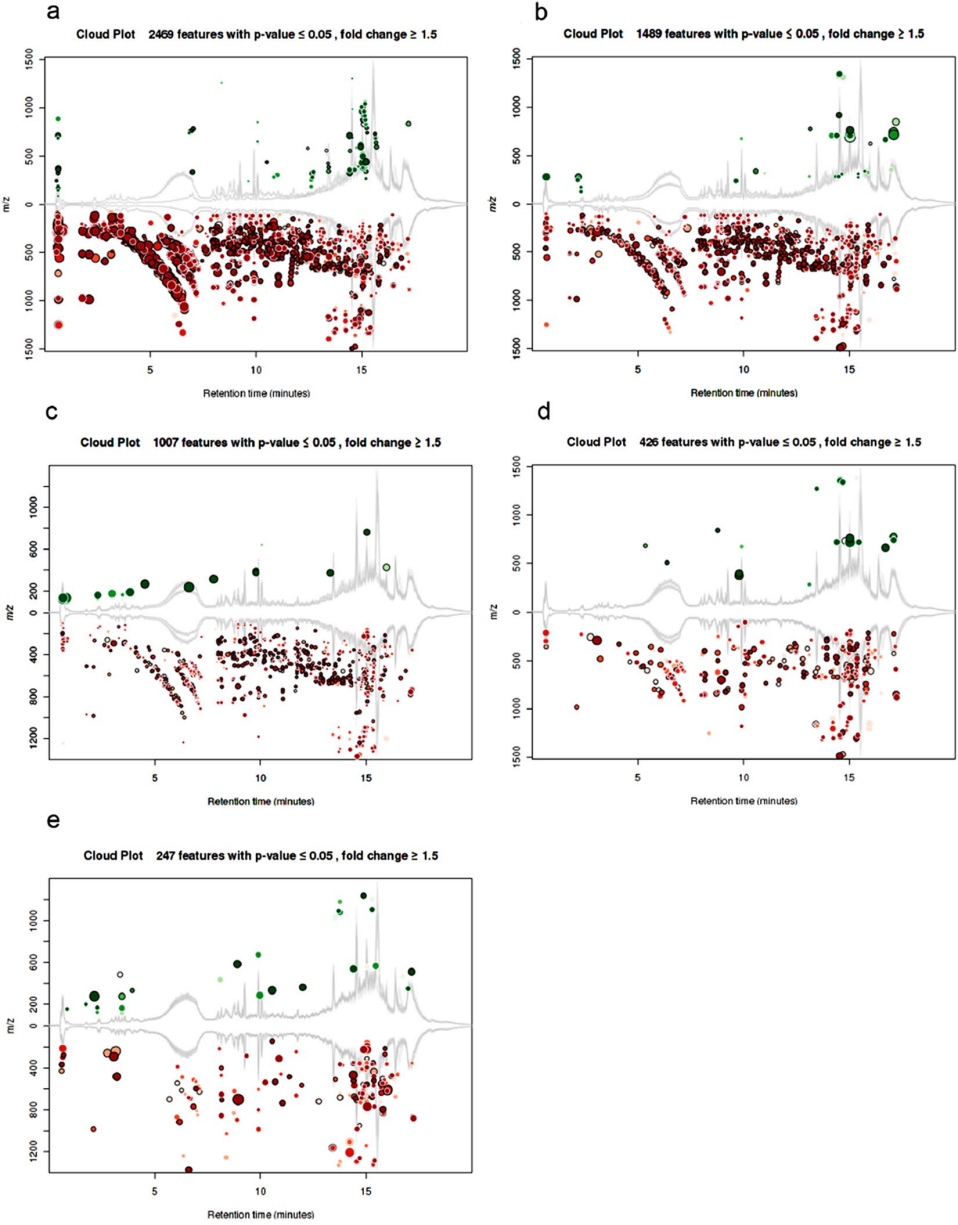
Cloud plot analysis of lipidomes of dormant and reactivated MTB showing the number and relative abundance of the features identified: normoxia vs dormancy (**a),** normoxia vs R24 (**b**), normoxia vs R48 (**c**), normoxia vs R72 (**d**) and normoxia vs R144 (**e**). Fold change >1.5; *p* < 0.05.

**Figure 3:**
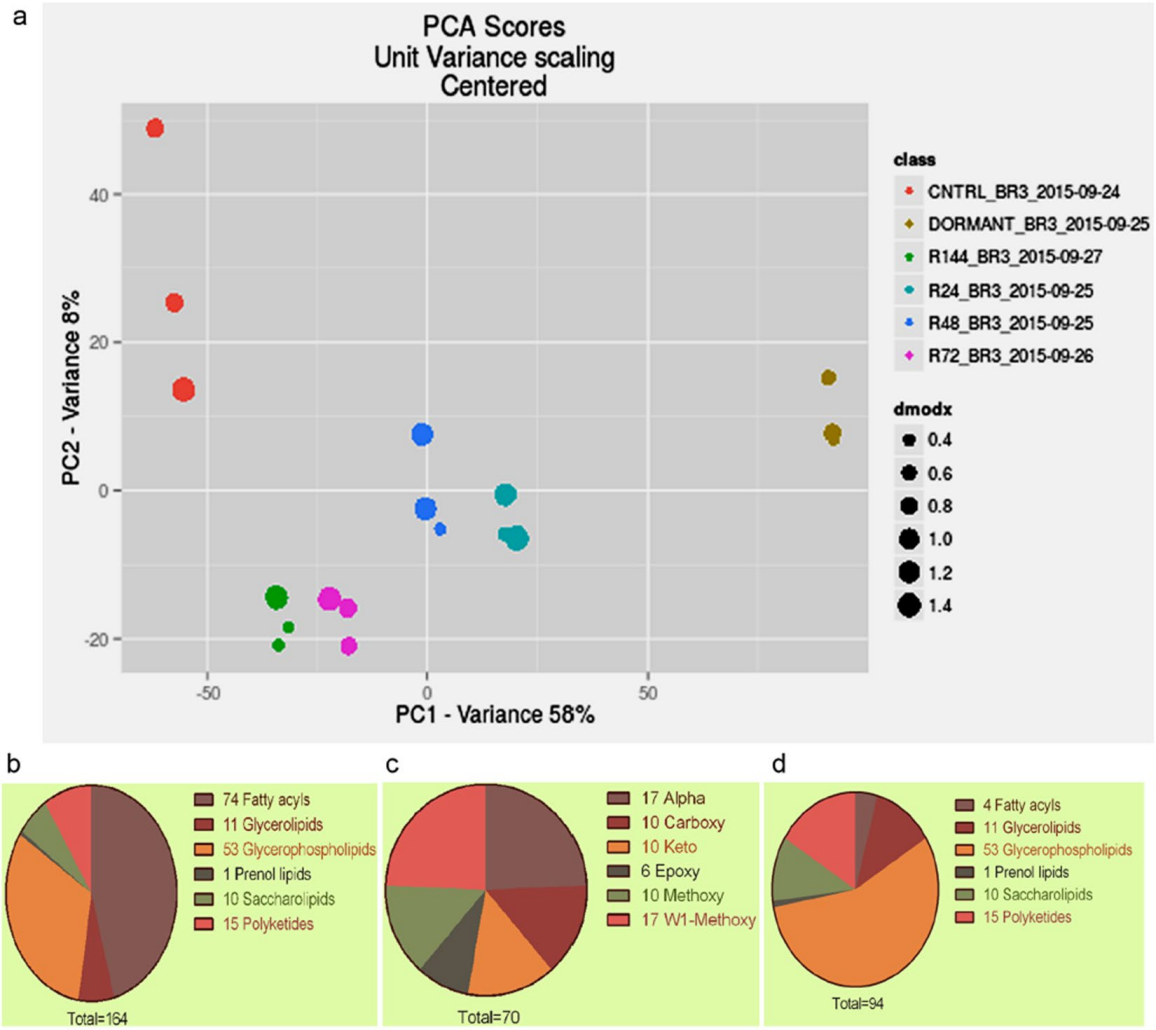
Principal component analysis (PCA) and grouping of the lipids identified. PCA plot of the features identified from lipidomes of dormant and reactivated MTB (**a**); representative plot from one of the three biological replicates. Categories of total lipids identified into various categories based on MycoMass database (**b**). The lipids were manually grouped into mycolic acids (**c**) and non-mycolic acids (**d**).

To identify the changes in lipid profile occurring on transition from normoxial growth to dormancy, and reactivation from dormancy, we analyzed the lipids from MTB at normoxia, dormancy, 24, 48, 72 and 144 h post re-aeration (R24, R48, R72 and R144). A lipid feature was defined as a three-dimensional value of *m/z,* retention time (RT) and intensity detected in triplicates. From all the conditions features with equivalent mass and retention time were aligned which enabled pair-wise comparison of MS signal intensity to analyze the lipids that were either present/absent or increased/decreased with a false positive rate below 1% (Supplementary Tables 1 and 2). This analysis yielded a total of 4187 significant features. During the transition from normoxial growth to dormancy 2480 features were found to have significant variation. As the cultures were re-aerated these differences decreased progressively, and at 144 h post re-aeration the variations with the normoxial features were minimum (247) which suggest that the bacteria have attained most of their normal features. Majority of the differentially manifested (normoxia vs dormancy) features were found to be low during dormancy (red dots) and the number of these features gradually decreased during the course of reactivation (Fig. 2a–e). In the principal component analysis (PCA) of the total features identified from each condition two of the reactivation stages (R72 and R144) were clustered together and were closer to the normoxial control with minimal variations (Fig. 3a). However, features of R24 and R48 were closer to each other whereas those of dormancy formed a distinct group with significant variations. Even though R24-associated features showed more similarity towards those of R48, a distinct resemblance to those of dormancy was evident. This is expected because at 24 h post re-aeration the bacteria have just started to revert from the dormant state, considering the long generation time of MTB. Among the features identified from all the four stages of reactivation those of R144 were closest to the features of normoxia suggesting that the bacteria have almost completely reverted to their normoxial physiological state.

### Identification and comparison of total lipidomes from normoxia, dormancy and reactivation

The individual *m/z* values from each of the conditions were processed using MarkerLynx to generate chemical structures and resulted in molecule identification with a mass accuracy of 5 ppm. The MycoMass database^20^ classifies mycobacterial lipids into six major categories - fatty acyls, glycerolipids, glycerophospholipids, prenol lipids, saccharolipids and polyketides. Under these categories we identified a total of 164 lipids that were present across all the experimental conditions (Fig. 3b). We classified them into mycolic acids (Fig. 3c) and non-mycolic acid lipids (Fig. 3d). Numbers of almost all classes of lipids were found to decrease drastically in dormant MTB, and gradually their levels reverted to normalcy during re-aeration. The status of each category of lipids observed during the three states is described below.

**Figure 4:**
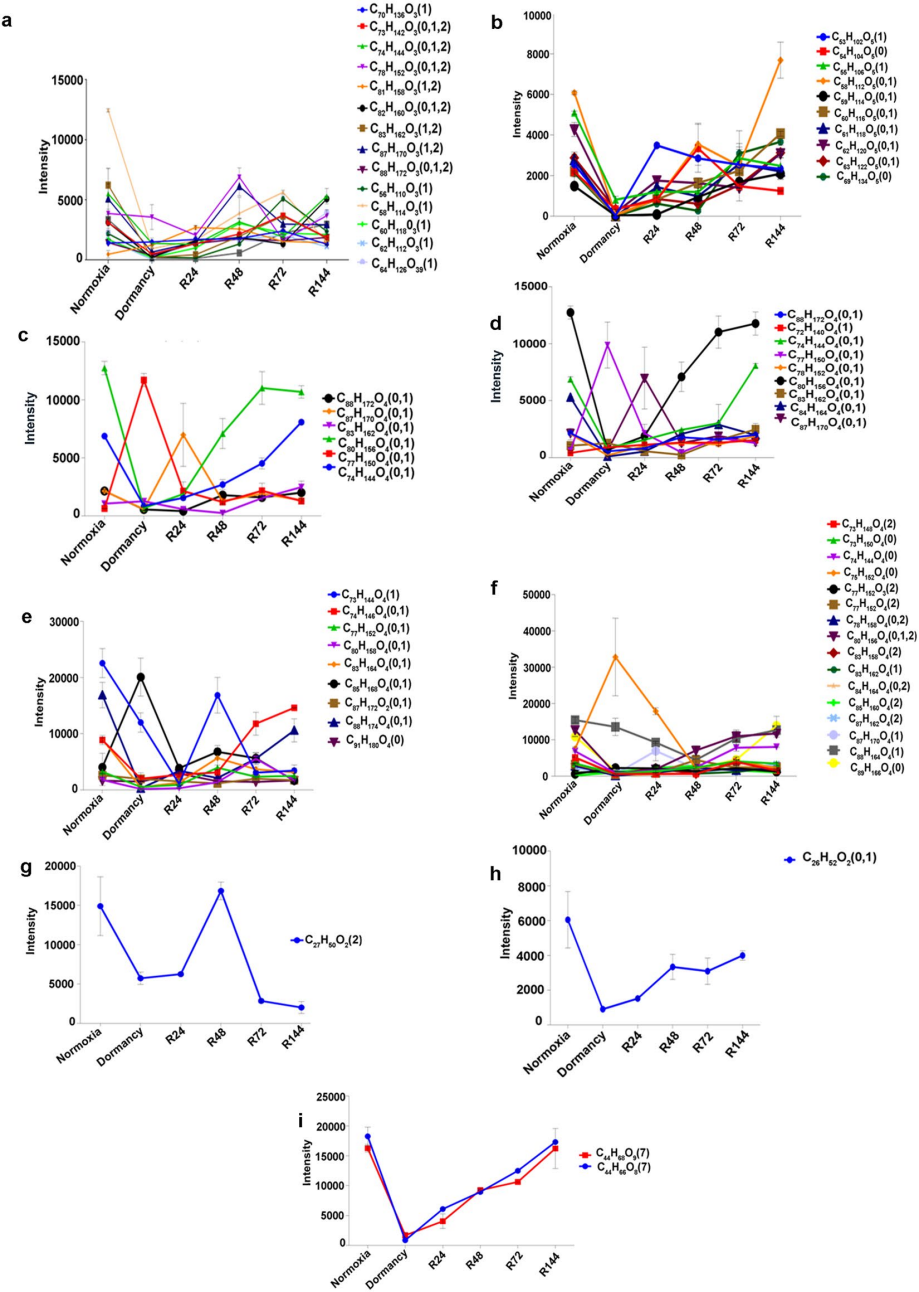
UPLC-MS analysis of total lipids from MTB in the positive-ion mode. The intensities of the 164 lipids identified, which were present across all the conditions analyzed, are plotted. Fatty acyls - mycolic acids: alpha (**a**), carboxy (**b**), epoxy (**c**), keto (**d**), methoxy(**e**) and W1-methoxy (**f**); mycolipodienic acid (**g**), mycoserosoic acid (**h**) and mycolactones (**i**). The elemental formulae and degree of unsaturation (in brackets) are given. Error bars represent standard deviations from three biological replicates.

**Figure 5:**
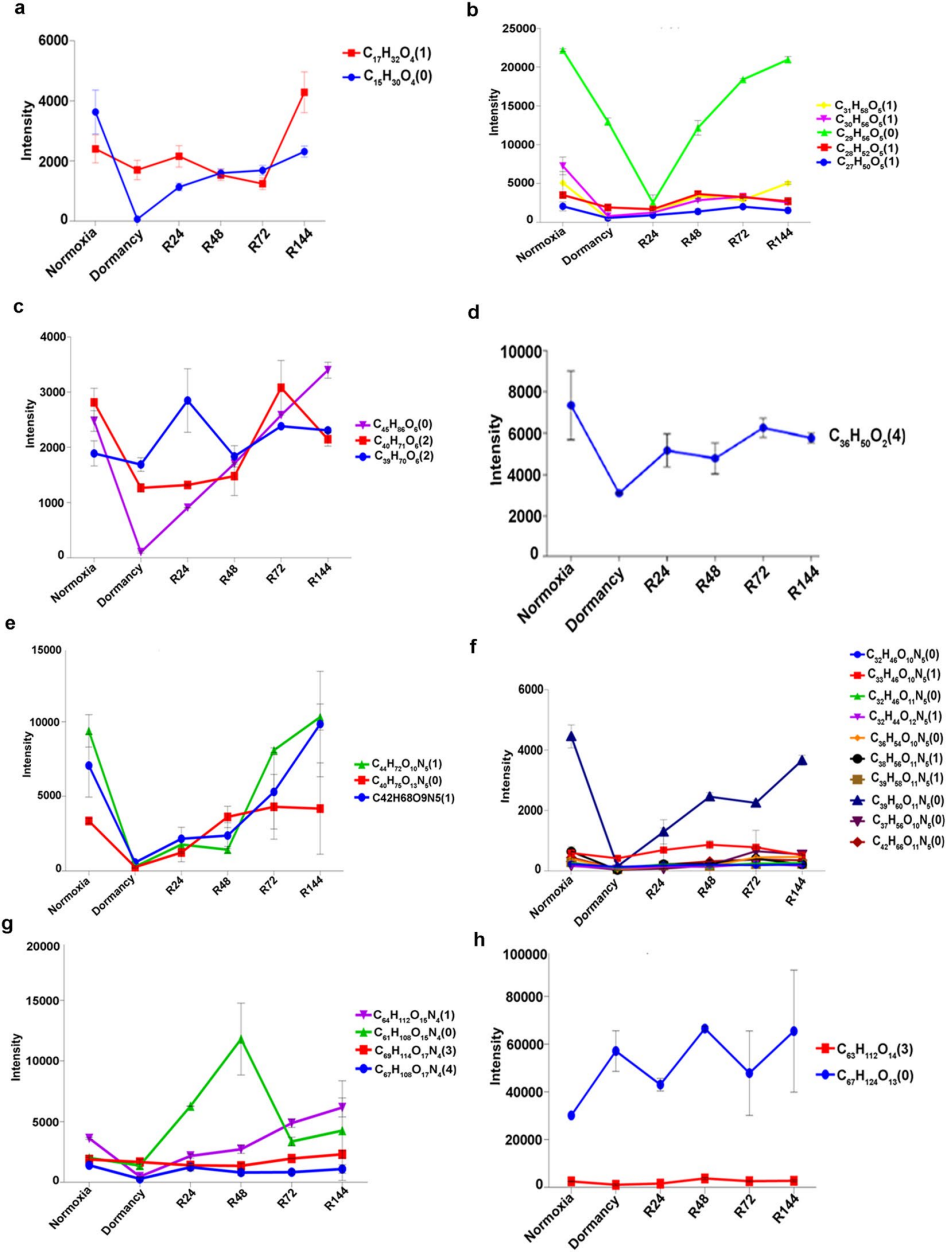
Intensities of members of glycerolipids: mono (**a**), di (**b**) and triacylglycerols (**c**); prenol: ubiquinone (**d**); polyketides: mycobactin (**e**), carboxymycobactin (**f**); saccharolipids: glycopeptidolipids (**g**) and diacyltrehaloses (**h**).

Fatty acyls include seven subclasses of lipids in the MycoMass database. We identified a total of 74 fatty acyls which showed significant variations across the three different states (normoxia, dormancy and reactivation) analyzed. Seventy of them belonged to mycolic acids. Among the mycolic acids we identified seventeen alpha forms, ten carboxy forms, six epoxy forms, ten keto forms, ten methoxy forms and seventeen W1-methoxy forms (Fig. 3c). Except for one (C_78_) all the other alpha mycolic acids were depleted during dormancy and these reverted to normalcy upon re-aeration (Fig. 4a). A similar trend was observed for the carboxy mycolic acids of which all were depleted during dormancy and reverted during re-aeration (Fig. 4b). In the epoxy group five were depleted during dormancy and they all reverted upon re-aeration. The same trend was observed in the keto group also where nine were depleted during dormancy and they reverted to normal levels during re-aeration. Interestingly in both epoxy and keto groups of mycolic acids, a C_77_ form was found to increase during dormancy and to decrease during the course of reactivation (Fig. 4cd). In the methoxy mycolic acid group nine forms increased during dormancy except for C_85_ (Fig. 4e). Under the W1-methoxy mycolic acid group 16 forms accumulated during dormancy except for C_75_ (Fig. 4f). The relative abundance of mycolic acids identified from all the conditions employing LC-MS analysis is shown in the form of a heat diagram (Supplementary Fig. 1). Other than mycolic acids we were able to identify one member each from mycolipodienic acid and mycocerosoic acid families (Fig. 4gh), and two from mycolactone family (Fig. 4i) all of which followed the general trend during dormancy and reactivation. We identified three families of glycerolipids i.e. mono, di and triacylglycerols across the different conditions. The two monoacylglycerolipids and five diacylglycerolipids were found to decrease during dormancy and they came back to normal levels upon aeration (Fig. 5ab). Triacylglycerols also followed the same pattern except for the C_39_ form which remained unaltered across all the three states (Fig. 5c).

In the class of prenol lipids, we were able to identify only one member of the menaquinone family which belongs to the ubiquinone subclass of lipids. Across all the conditions tested this menaquinone followed the same pattern as observed in other classes of lipids (Fig. 5d). Two families of mycobacterial lipids that belong to polyketide category were identified which included mycobactin and carboxymycobactin families. We observed three types of mycobactins (C_44_, C_40_ and C_42_) all of which were depleted during dormancy and regained normal levels during the course of reactivation (Fig. 5e). A total of 10 carboxymycobactins were identified of which the C_39_ form was depleted during dormancy and reverted to normal levels during reactivation. Interestingly the other nine carboxymycobactins did not show much variation during dormancy and reactivation (Fig. 5f). Among the saccharolipid category we could identify glycopeptidolipids and diacyltrehalose families. Strikingly there were no significant changes in the profiles of these groups under normoxia, dormancy and reactivation (Fig. 5gh).

**Figure 6:**
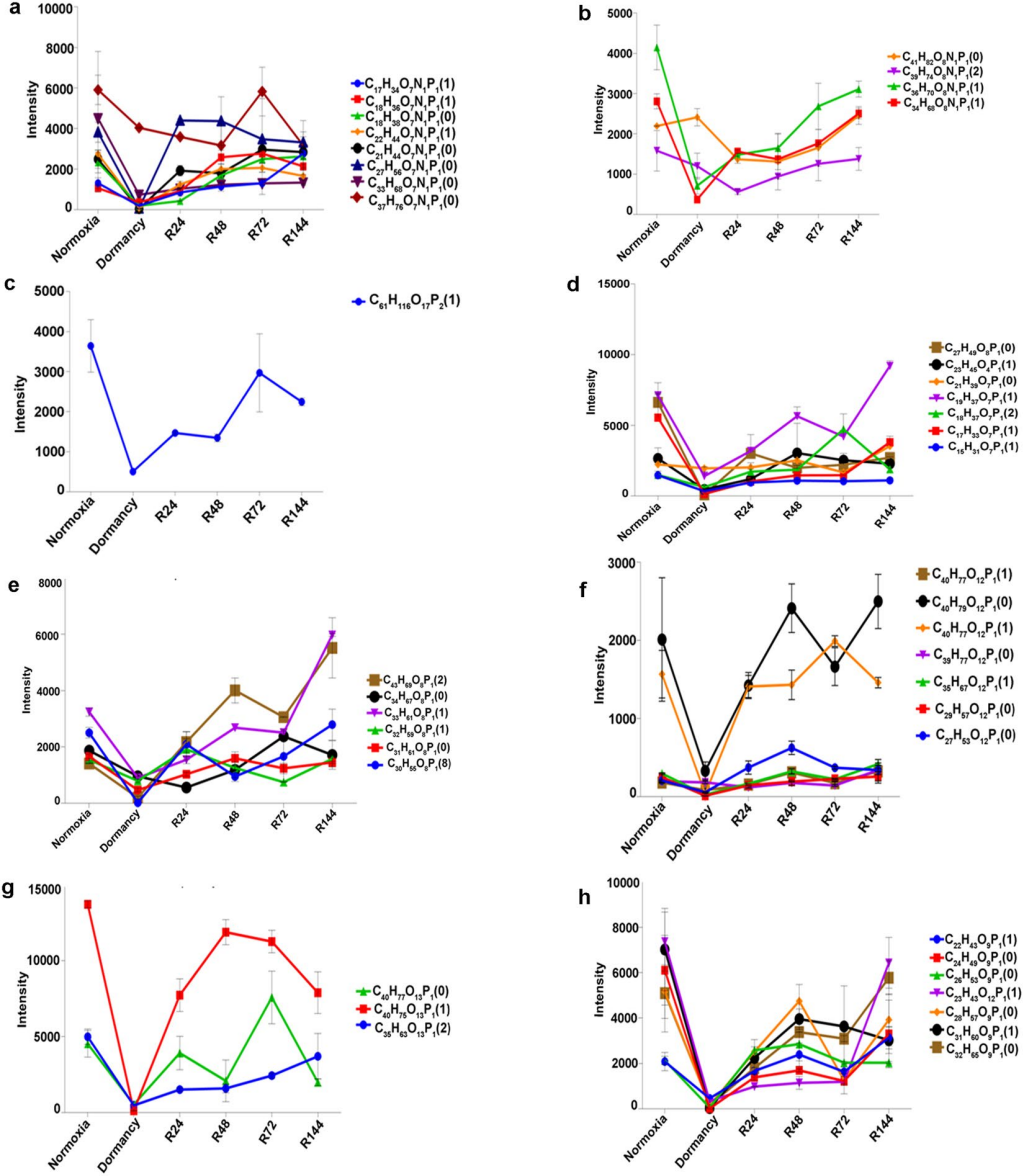
Intensities of various glycerophospholipids: lysophosphatidylethanolamines (**a**), phosphatidylethanolamines (**b**), cardiolipin (**c**), lysophosphatidic acids (**d**), phosphatidic acids (**e**), lysophosphatidicinositols (**f**), phosphatidylinositols (**g**) and lysophosphatidylglycerols (**h**) are shown.

Glycerophospholipids form the largest class of mycobacterial lipids with thirteen subclasses. Majority of these lipids are associated with bacterial membrane^22^. We identified eight lysophosphatidylethanolamines, four phosphatidylethanolamines, one cardiolipin, seven lysophosphatidic acids, six phosphatidic acids, seven lysophosphatidylinositols, three phosphatidylinositols and seven lysophosphatidylglycerols across all the three states of MTB analyzed (Fig. 6a–h). All these lipid families followed the trend of decreasing drastically during dormancy and reverting to normoxial levels during reactivation.

### Mycolic acids are depleted rapidly during dormancy

Mycolic acids, the main components of the cell envelope of MTB, are 2-alkyl 3-hydroxy long-chain fatty acids^23,24^. We attempted to examine the variations in the mycolic acid content using HPLC analysis of derivatized mycolic acids from normoxially grown, dormant and reactivated MTB. The chromatogram of mycolic acids from normoxially grown bacteria showed a characteristic pattern with seven distinct peaks along a 20 min gradient run (Supplementary Fig. 2a). On the other hand, the chromatogram of dormant mycobacteria was strikingly different showing only one peak (Supplementary Fig. 2b). At 24 h post re-aeration the missing peaks started to reappear which indicated that the depleted mycolic acids were in the process of being synthesized (Supplementary Fig. 2c).

**Figure 7:**
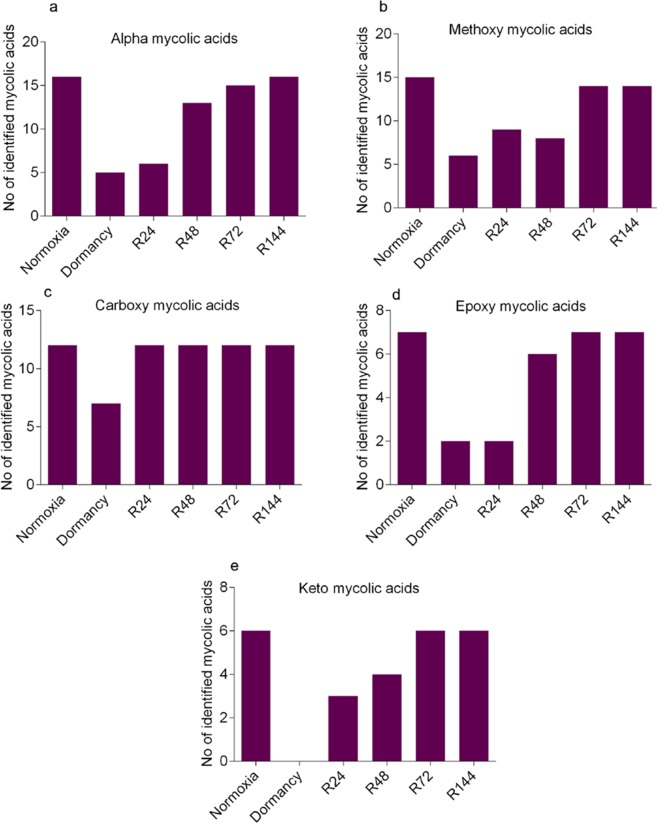
MALDI-TOF/MS analysis of mycolic acids from dormant and reactivated MTB. Mycolic acids were isolated from MTB subjected to dormancy and re-aeration, and MALDI-TOF/MS in positive mode was used to generate the *m/z*. The masses were matched with MycoMass database to identify classes of mycolic acids. Numbers of mycolic acids identified under various conditions are depicted: alpha (**a**), methoxy (**b**), carboxy (**c**), epoxy (**d**) and keto (**e**).

To discover the classes of mycolic acids that are altered during dormancy and reactivation we performed a MALDI-TOF/MS analysis of the total mycolic acids across all the conditions. This analysis yielded a set of masses which were matched with the MycoMass database. Similar to what we found in the LC-MS analysis, we observed considerable depletion of different types of mycolic acids during dormancy and its subsequent reappearance during the course of re-aeration. A reduction in four forms of mycolic acids (alpha, methoxy, carboxy and epoxy) was observed under dormancy (Fig. 7a–d). Noticeably no keto form was detected under dormancy although it was detected by the LC-MS analysis (Fig. 7e). We assume it is due to the lesser sensitivity of the MALDI-TOF/MS detection system. However the mycolic acids (alpha, methoxy and keto) identified from normoxia and R144 were found to be exactly the same by both the mass spectrometric methods employed.

## Discussion

The phenomenon of dormancy is a highly complex adaptation by MTB to survive the challenging environment inside the host^2,3^. The bacteria are walled off inside structures called granuloma where the bacteria are exposed to different stress conditions such as nutrient starvation, hypoxia, acidic and oxidative/nitrosative stress, etc. Under these conditions MTB is known to undergo drastic changes in its energy and metabolic status^25^. A number of studies have highlighted the importance of lipid metabolism during the transition from dormancy to reactivation^26,27^. In this study we analyzed lipids of MTB under three different simulated states namely normoxial growth, dormancy and re-aeration. From 4187 features resolved by retention time and mass-to-charge ratio (*m/z*) we were able to identify a total of 164 lipids from all the three conditions analyzed. These lipids were clustered into six major categories and were fine-mapped to their respective families in accordance with the level 4 classification^20^. MTB uses beta oxidation of fatty acyls to persist within the host^18^. Even-chain fatty acids are catabolized to acetyl CoA and odd-chain fatty acyls are converted to propionyl CoA. Chemotaxonomic data on the length of alkyl side-chain and the degree of unsaturation of fatty acyls coupled with their signal intensities suggest evidence of lipid degradation by beta oxidation during dormancy.

MTB stores fatty acids in the form of triacylglycerol (TG) for countering adverse situations like hypoxia and nutrient starvation^28^. Significant changes in cellular concentrations of TG occur when the bacterial culture progresses from logarithmic to stationary phase; the highest amount being present in stationary cultures^19^. Galagan *et al*. (2013) reported accumulation of TGs (<C_60_) during hypoxia and their rapid depletion during re-aeration^15^. However we did not observe this phenomenon. This is perhaps due to the fact that TGs that we detected were of short chain length (<C_50_) which were not accumulated by the bacterium during dormancy.

Mycolic acids are the major lipid components of the cell envelope of MTB and are critical determinants of the mycobacterial physiology during dormancy^29^. Our LC-MS as well as MALDI-MS analysis revealed a marked decrease in all the forms of the mycolic acids in dormant MTB. Conversely, Galagan *et al*. (2013) found an increase in free mycolic acids during dormancy and these were reversed during reactivation^15^. We speculate that the difference is attributable to a late onset of dormancy achieved in our model. Many studies have revealed that dormant bacteria do not induce an immune response^30^. One of the reasons for this phenomenon could be the depletion of mycolic acids, which are TLR activators from the cell wall of the bacteria, rendering them incapable of eliciting an immune response. Our SEM analysis revealed that the surface properties of dormant MTB were compromised and the length of the bacilli was reduced considerably during dormancy. However once they were subjected to aeration the bacteria began to regain their original morphology, and at 144 h post re-aeration the morphology was indistinguishable from that of normoxially grown bacteria.

It is interesting to note that some lipids like C_77_H_15_O_4_ and C_85_H_168_O_4_ were abundant or showed an increase under dormancy. Although we are unable to provide an explanation for this phenomenon, it, however, offers the possibility that these lipids can be exploited as diagnostic markers to distinguish between dormant and active TB. Further studies are required in this direction. During re-aeration intensities of some lipids such as C_74_H_144_O_4_ (R144) C_74_H_146_O_4_ (R72) were higher than those of normoxially grown MTB. This could be due to enhanced *de novo* lipid biosynthesis during re-aeration which could stabilize during prolonged normoxial growth. The fact that levels of certain lipids of dormant MTB increased beyond those of normoxially grown cells suggests that regaining of cellular morphology upon re-aeration may not necessarily correlate with the regaining of cell wall composition. Like all other *in vitro* models of dormancy our model also falls short in mimicking the natural *in vivo* conditions in the granulomas. Inside granulomas MTB metabolizes host lipids efficiently^31,32^. Nonetheless our study provides an insight into the possible impact of hypoxia and resuscitation on lipid metabolism of MTB. Given the complexity of the MTB lipidome, detecting as well as identifying them from granuloma models offer a huge technical challenge. Detailed pathway analyses involving transcriptomic, proteomic and metabolomics approaches are required to determine if the mechanisms regulating lipid signatures and their levels observed in MTB in our dormancy and resuscitation model are similar to or different from those in the granuloma.

## Materials and Methods

### Bacterial culture conditions

*M*. *tuberculosis* virulent laboratory strain H37Rv was subcultured on Löwenstein-Jensen slants and incubated at 37 °C for 4–6 weeks. All steps involving handling of MTB were carried out in a biosafety level three (BSL3) facility. Broth cultures were prepared by inoculation of one loopful of bacterial colony into Dubos broth base (Difco, Franklin Lake, NJ) containing 5% (v/v) glycerol supplemented with Dubos albumin (2% v/v Difco). Culture grown to A_600_ of 0.6 (∼10^8^ bacteria per ml) in 250 ml conical flasks on a shaker incubator at 150 rpm at 37 °C was used as the inoculum. Bacteria were made dormant following protocol described by Gopinath *et al*.^16^. Briefly, aliquots of Dubos Tween-albumin broth containing 2 × 10^6^ bacteria per milliliter were dispensed into flat-bottomed glass tubes. Cultures were grown with limited internal agitation of 130 rpm using 8 mm Teflon-coated magnetic bars (Sigma-Aldrich St. Louis, MO) on multipoint magnetic stirrers (Variomag Poly 15, Thermo Scientific, Waltham, MA). The cap (Pressure compensation set, Duran, Germany) of the glass tube was connected to a 0.2 micron filter using a silicon tubing (3 cm length and 1.6 mm internal diameter). The tubing was kept airtight using pinchcock clamp. The whole setup was placed inside a custom-made 37 °C incubator (Santhom Scientific, Bangalore, India). The dormant bacteria were re-aerated by removing the pinchcock clamp from the silicone tubing and increasing agitation to 200 rpm to facilitate aeration. The status of self-generated hypoxia and reactivation was monitored visually using methylene blue. Growth was monitored every 24 h by measuring A_600_ in a colorimeter (Aimil Photochem, New Delhi, India).

### Electron microscopy

Twenty milliliters of culture from different conditions was centrifuged at 5000 g for 10 min at 25 °C. The pellets were then washed thrice in 10 ml ice-cold PBS and centrifuged at 5000 rpm for 5 min at 25 °C. Pellets were dissolved in 1 ml Dubos Difco broth and later vortexed vigorously to disperse bacterial clumps. Suspensions with an OD (600 nm) of 0.15 were prepared in fresh Dubos broth. Three hundred microliters from each sample was then loaded onto polylysine-coated cover slips that were prepared according to manufacturer’s guidelines (Sigma-Aldrich St. Louis MO USA). Bacteria were allowed to settle for 30 min before gently decanting the medium. One milliliter of 2.5% glutaraldehyde in PBS (pH 7.4) was added to the bacterial film on the coverslip and incubated overnight at 4 °C. A series of dehydration steps were performed for 10 minutes each in 50%, 70%, 95% and 100% ethanol and the coverslips were dried at room temperature. Samples were gold-sputtered using JEOL-1200 (Peabody, MA, USA) and imaged using a JEOL-JSM-5600 LV scanning electron microscope.

### Isolation of lipids from normoxially grown, dormant and reactivated *M*. *tuberculosis* H37Rv

MTB under various states (normoxia, dormant and re-aerated at 24, 48, 72 and 144 h (R24, R48, R72 and R144)) were centrifuged (5000 g, 10 min, 25 °C). Cell pellets were washed twice in sterile water (10 ml) centrifuged for 10 min at 4000 g at 25 °C. Four hundred milligram of pellet from each condition was then resuspended in 1 ml of methanol, transferred to a 50 ml tube and added 25 ml of chloroform: methanol (2:1 v/v) and was incubated overnight. The suspensions were shaken on a rotary shaker (Orbitron Basel Switzerland) for at least 1 h at 25 °C after overnight incubation. After centrifugation at 4000 rpm for 10 min at 25 °C the lipid extracts were transferred to a new tube and the bacterial pellets were subjected to two additional extractions using chloroform: methanol (1:1 and 1:2 ratios, v/v). Internal standards phenylalanine (1 µg/ml in water), reserpine (1 µg/ml in methanol) and sulfadimethoxine (1 µg/ml in methanol) were added to the samples prior to extraction. All extracts were dried under nitrogen gas. One milligram of dried lipid was resuspended in 1 ml methanol and used for further analysis.

### LC-MS ANALYSIS

#### Liquid Chromatography

Analysis was performed using a Waters ACQUITY UPLC^TM^ system (Waters, Milford, MA, USA) coupled to a Quadrupole-Time of Flight (Q-TOF) mass spectrometer (SYNAPT-G2 Waters). Both the systems were operated and controlled by MassLynx4.1 SCN781 software (Waters). The lipidomic separation was achieved using reverse-phase liquid chromatography employing a C18 (high-strength silica 2.1 × 100 mm, 1.8 µm; Waters) column. Briefly, a 7.5 µl aliquot of each sample was injected into the column with the column temperature maintained at 40 °C. For each sample the run time was 20 min with a flow rate of 400 µl/min. The mobile phase consisted of aqueous (A) and organic (B) solvent components where A was 0.1% formic acid in ultrapure water and B was 0.1% formic acid in acetonitrile. The gradient was 0 min 1% B; 2 min 10% B; 6 min 30% B; 8 min 50% B; 12 min 75% B; 15 min 99% B and 20 min 100% B. Each sample was injected in triplicate with blank injections between each sample.

### Mass spectrometry

The SYNAPT® G2 High Definition MS™ System mass spectrometer (Waters) was operated in positive resolution mode with electrospray ionization (ES^+^) source over a mass range of 50 to 1500 Da. Each spectrum was acquired for 0.25 sec with an interscan delay of 0.024 sec. Ion source and desolvation gas (nitrogen) temperatures were kept at 130 °C and 450 °C, respectively. The cone and desolvation gas flow rates were 80 L/h and 600 L/h, respectively. Capillary voltage was set at 3.0 kV. Sampling cone voltage was set at 30 kV. The lock mass acquisition was performed every 30 sec by leucine-enkephalin (556.2771 [M + H]^+^) for accurate on-line mass calibration. The data acquisition was carried out in centroid mode and processed using MassLynx software.

### XCMS analysis and quality control

The raw datasets from each condition were processed by XCMS integrated metabolomics analysis platform for filtering noise peak detection and deconvolution to resolve coeluting ions and peak alignment across replicates. The features with identical AMRT values were aligned across biological conditions and their intensities were generated as a data matrix^33^. A feature was defined as a three-dimensional value of *m/z* retention time (RT) and intensity detected in triplicate.

Ceramide/Sphingoid Internal Standard Mixture II (Avanti Polar Lipids, Alabaster, AL, USA) was used as quality control to check the efficiency and quality of mass spectrometry runs. Quality control mix was run after every 10 sample runs. To ensure normalization of the input we incorporated three internal standards in the LC runs. The difference in intensities of reserpine and sulphadimethoxine were found to be well within the accepted limits (a maximum of 10% variation) between biological replicates (Supplementary Fig. 3ab) as per Layre *et al*.^20^. Run normalizations using Ceramide/Sphingoid Internal Standard mixture II (CSIS-II) were also performed and we detected 5 of the 9 standards (with 1–3% variation) in the biological replicates (Supplementary Fig. 4a–e).

### Data processing

MassLynx4.1 SCN781 (Waters) was used for data acquisition and collection. Markerlynx XS software (Waters) was employed for peak/feature picking and raw data de-convolution, noise filtering, peak detection, isotope peak removal, alignment of retention time and mass as well as optional peak/feature normalization. Method parameters for Markerlynx processing were as follows: peak width at 5% height - 1 S; marker intensity threshold - 10 counts; mass window - 0.05 Da and retention time window - 0.2 min. Automatic naming of compound was performed whose *m/z* matched within 5 ppm range and further validated by comparing the *m/z* with MycoMass and MTB Lipid DB databases.

### Isolation of mycolic acids from *Mycobacterium tuberculosis* H37Rv

Mycolic acids were isolated from MTB by the protocol recommended by the Centre for Disease Control (CDC)^34^. Mycobacteria from various states (20 ml from normoxia, 80 ml from dormancy and R24) were centrifuged at 5000 rpm for 10 min at 25 °C washed twice in 10 ml of ice-cold PBS. To 400 mg of the pellet 2 ml of saponification reagent (KOH 2 g, reagent- grade water 400 ml, methanol 400 ml) was added and the mixture was transferred to a glass screwcap tube. The contents of the tubes were mixed vigorously on a vortex mixer. The lids were covered with an aluminum foil and the tubes were heated for 2 h at 100 °C on a heating block. The tubes were subsequently allowed to cool to 25 °C. Two milliliters of HPLC-grade chloroform and 1.5 ml of acidification reagent (reagent-grade water 400 ml, concentrated HCl 400 ml) were added to the contents of the tubes and mixed vigorously on a vortex mixer. The tubes were allowed to stand to separate the aqueous and chloroform layers. The bottom chloroform layer was removed and transferred to a new glass screwcap tube. The chloroform was removed by flushing with nitrogen gas. One hundred microliters of potassium bicarbonate reagent (KHCO_3_ 4 g, reagent-grade water 98 ml and methanol 98 ml) was added to 2 mg of mycolic acids and was evaporated on a heating block at 85 °C under a stream of nitrogen. The samples were cooled to room temperature and 1 ml of chloroform was added followed by 50 µl of derivatization reagent (p-bromophenyl acyl bromide 0.1 mmol/ml and dicyclohexyl-18-crown-6-ether 0.005 mmol/ml in acetonitrile). The tubes were capped tightly and mixed vigorously. The samples were again heated on a heating block at 85 °C for 30 min. After cooling the samples to room temperature one milliliter of clarification reagent (100 ml of acidification reagent mixed with 100 ml of methanol) was added and mixed. The layers were allowed to separate and the bottom chloroform layer was removed and transferred to a new glass tube. The chloroform was flushed out using nitrogen gas and 1 mg of derivatized mycolic acids was weighed from each sample and dissolved in HPLC-grade dichloromethane to enable their detection by UV.

### HPLC analysis

HPLC was performed using a C18 column (Waters, 10 µm pore size 4.6 × 300 mm). The solvent flow rate was maintained at a constant flow of 2.5 ml/min for all the samples. The initial solvent mixture was 98% methanol (solvent A) and 2% dichloromethane (solvent B) (98:2 v/v). During the first one minute following injection the solvent mixture was 80% A and 20% B using a linear gradient (elapsed time 1 min) (80:20 v/v). During the next 9 min the solvent mixture was changed to 35% A and 65% B using a linear gradient (elapsed time 10 min) (35:65 v/v). During the next 0.5 minute the gradient was returned to 98% A and 2% B (elapsed time 10.5 min) (98:2 v/v). Finally the solvent mixture was held at 98% A and 2% B to equilibrate the column for a duration of 5 min (total elapsed time 15 min) (98:2 v/v).

### MALDI-TOF/MS analysis

The mycolic acids extracted from MTB were analyzed on UltrafleXtreme (Bruker Daltonics, Billerica, MA, USA) equipped with smart beam solid state laser (337 nm) in reflectron positive ion mode using 19 kV acceleration voltage. The samples were co-crystallized with DTH matrix on the target plate (384-well ground steel plate, Bruker Daltonics) and external peptide mass calibration was applied (Peptide mixture 1700–3500 *m/z*) as per the manufacturer’s instructions. The MS data was acquired in the mass range of 700–3500 *m/z*. The mass spectra were imported from flux control to flux analysis for further processing.

### Statistical analysis

Principal component analysis was performed using XCMS software. To analyze the significance of changed features and assignment of *p* values nonparametric Bonferroni test was employed for all the lipids identified in the three biological replicates using GraphPad Prism V6 (La Jolla CA USA).

## Supplementary information

Supplemental info
