# Supplementary material for: Comparative label-free lipidomic analysis of Mycobacterium tuberculosis during dormancy and reactivation
Source: Sci Rep. 2019 Mar 6;9:3660. doi: 10.1038/s41598-019-40051-5 (PMC6403389; doi:10.1038/s41598-019-40051-5)
Supplement: Supplementary file 1 — Supplemental info [file 41598_2019_40051_MOESM1_ESM.pdf]

Comparative label-free lipidomic analysis of *Mycobacterium tuberculosis* during dormancy and reactivation.

Sajith Raghunandanan[1#], Leny Jose[1#,], Vipin Gopinath[1], Ramakrishnan Ajay Kumar[1].[*]

Supplementary Figures:

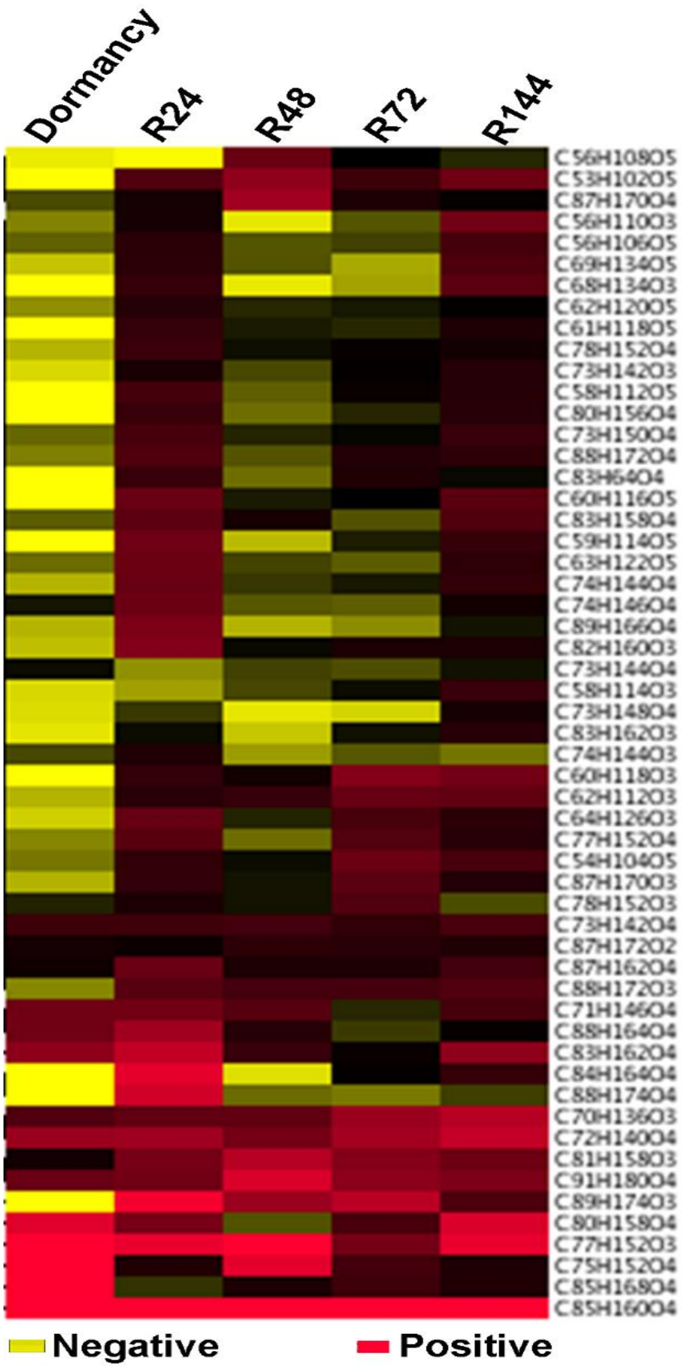

Figure S1: Heat map showing relative fold expression of mycolic acids identified from LC-MS analysis across various conditions. Positive values indicate elevated mycolic acids, and negative values represent depleted mycolic acids.

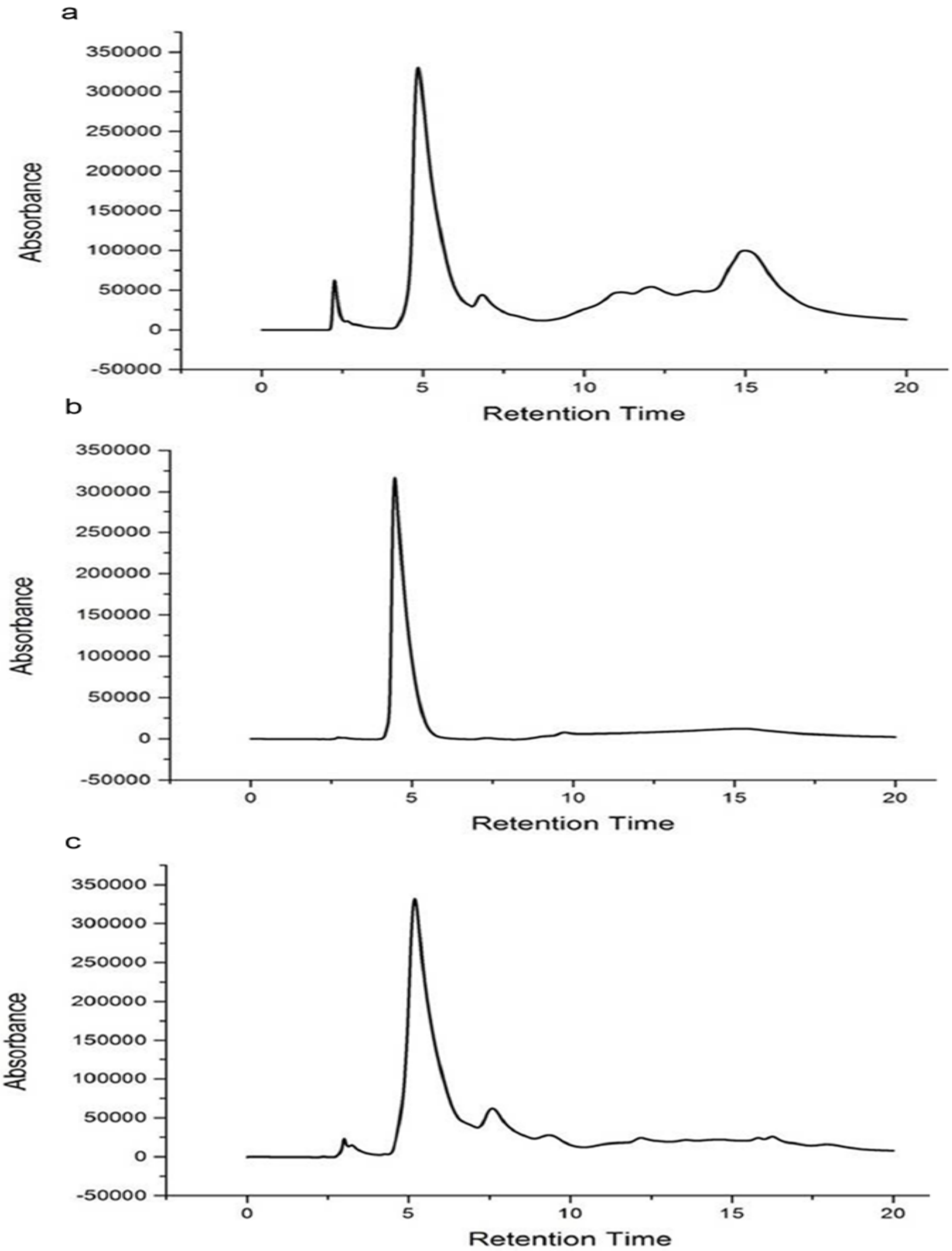

Figure S2: HPLC analysis

Chromatograms of HPLC analysis of mycolic acids isolated from MTB under normoxia (a), dormancy (b) and R24 (c) stages. X and Y axes indicate retention time and absorbance, respectively.

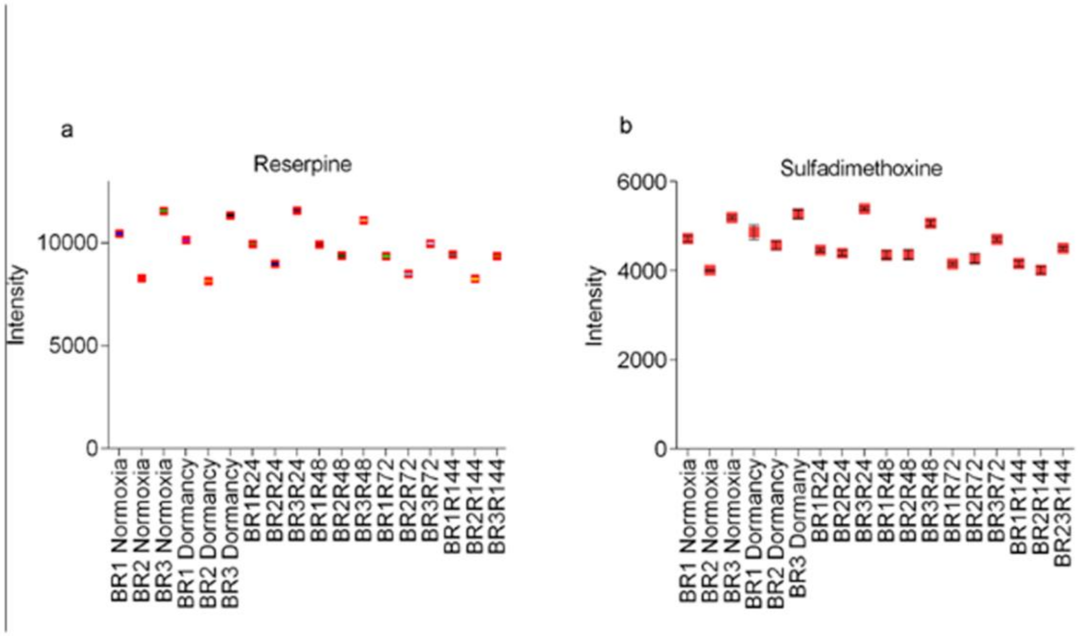

Figure S3: Sample normalization

Scatter plots showing values of internal standards (a) reserpine and (b) sulfadimethoxine, after post run analysis across each biological replicates and conditions- normoxia, dormancy, R24, R48, R72 and R144. Error bars represent standard deviations from three technical replicates of each biological condition.

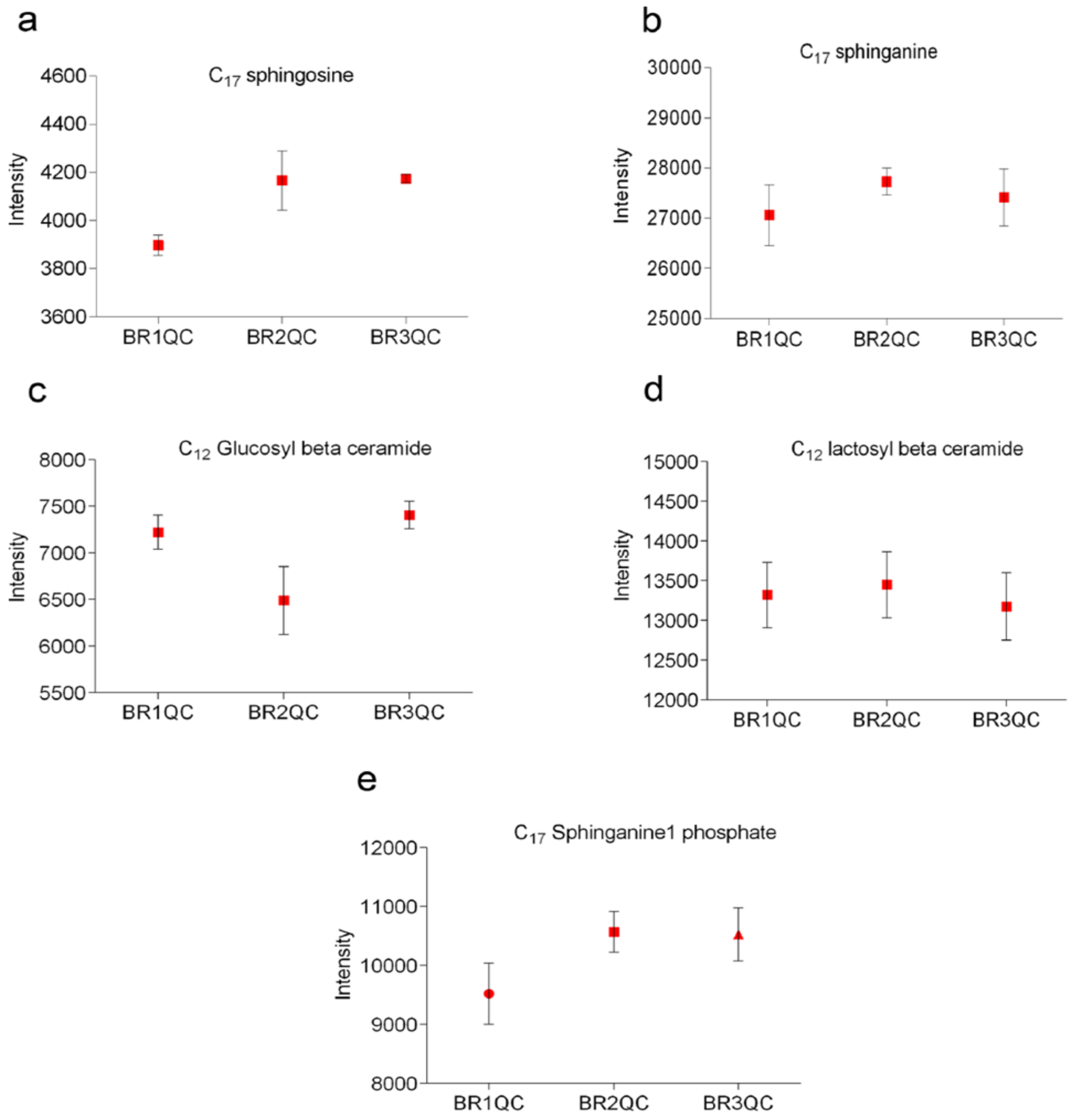

Figure S4: Run normalization

Mass spectrometry runs were normalized using Ceramide/Sphingoid Internal Standard mixture II, which are represented as scatter plots C_{17} sphingosine (a), C_{17} sphinganine (b), C_{12} glucosyl beta ceramide (c) C_{12} lactosyl beta ceramide (d) and C_{17} sphinganine-1-phosphate (e) across three

biological replicates. Error bars represent standard deviations from three technical replicates of each biological condition.

Sl No	Compond	Elemental composition	Normoxia (Control)	Dormancy	R24	R48	R72	R144
1	dimycocerosates	$C_{61}H_{116}O_{17}P_2$	2462.801	493.5462222	1479.312778	1762.606667	1610.577222	2257.301222
2	carboxymycobactins	$C_{32}H_{46}O_{10}N_5$	3833.902	1565.286667	2179.787222	1762.342111	1299.852333	2658.384556
3	carboxymycobactins	$C_{33}H_{46}O_{10}N_5$	14365.401	5307.834778	6025.324667	7679.941111	3909.229778	4169.822667
4	carboxymycobactins	$C_{32}H_{46}O_{11}N_5$	3268.448	1154.810111	1920.717222	1645.816667	1813.897	2884.194111
5	carboxymycobactins	$C_{32}H_{44}O_{12}N_5$	2089.243333	733.5842222	1397.511667	1363.075889	3140.932333	1897.660778
6	carboxymycobactins	$C_{36}H_{54}O_{10}N_5$	4379.602	750.4908889	1746.358778	1967.135556	3429.945	3786.276556
7	carboxymycobactins	$C_{38}H_{56}O_{11}N_5$	5095.502833	384.7973333	1944.835667	2782.031222	3388.206444	3104.342
8	carboxymycobactins	$C_{39}H_{58}O_{11}N_5$	2827.453667	404.5922222	1529.856333	1630.944778	2015.578667	2513.655778
9	carboxymycobactins	$C_{39}H_{60}O_{11}N_5$	39526.09233	1786.482556	9090.886222	23333.46167	24848.31522	34722.56556
10	carboxymycobactins	$C_{37}H_{56}O_{10}N_5$	1789.566167	1211.010222	1090.271	1555.734333	4591.460111	2519.458111
11	carboxymycobactins	$C_{42}H_{66}O_{11}N_5$	6032.489833	439.5536667	2721.299556	2543.734111	3175.473222	5060.132111
12	cardiolipin	$C_{61}H_{116}O_{17}P_2$	2434.314667	394.1804444	1248.285111	1535.340556	2870.696333	2091.757111
13	diacylglycerols	$C_{27}H_{50}O_5$	2823.676667	470.0637778	1014.091	1917.091556	1740.809222	1756.185889
14	diacylglycerols	$C_{28}H_{52}O_5$	4213.823833	2360.419222	1400.253556	3279.441111	3135.675	2864.035667
15	diacylglycerols	$C_{29}H_{56}O_5$	35432.907	16424.79656	5684.412889	23280.57744	22374.896	21470.30289
16	diacylglycerols	$C_{30}H_{56}O_5$	5444.414667	1673.153333	2728.436778	3297.753778	3062.046111	3130.903222
17	diacylglycerols	$C_{31}H_{58}O_5$	5062.217167	346.8801111	1641.236556	3925.796778	3364.164556	4356.517222
18	diacyltrehaloses	$C_{36}H_{66}O_{13}$	4038.079333	474.3426667	1761.721556	2570.487889	3893.207	3607.865111
19	diacyltrehaloses	$C_{36}H_{54}O_{10}N_5$	12317.939	326.7103333	2932.326778	9166.317889	7641.573	11487.81744
20	diacyltrehaloses	$C_{37}H_{68}O_{13}$	29229.31417	1108.295778	31134.28178	21316.55378	17991.7	44801.17511
21	diacyltrehaloses	$C_{45}H_{80}O_{13}$	5515.684333	264.1428889	3726.849778	5119.260333	5720.475778	4088.242889
22	glycopeptidolipids I	$C_{67}H_{108}O_{17}N_4$	1409.493167	219.0932222	1330.28	1093.449778	1069.788556	1378.661556
23	glycopeptidolipids I	$C_{69}H_{114}O_{17}N_4$	1891.980333	1407.808222	1483.742111	1495.055111	1994.993667	2081.494778
24	glycopeptidolipids IIa/IV	$C_{61}H_{108}O_{15}N_4$	7123.474	4022.169333	5609.677333	8778.807222	7208.926778	9169.259333
25	glycopeptidolipids IIa/IV	$C_{64}H_{112}O_{15}N_4$	4978.9605	467.8326667	1901.505556	3941.552333	4090.154111	5046.662444
26	lyso phosphatidic acids	$C_{15}H_{31}O_7P_1$	1274.867667	367.7856667	973.0113333	1191.964556	1223.940667	1292.085111
27	lyso phosphatidic acids	$C_{17}H_{33}O_7P_1$	4266.948333	203.1305556	883.7825556	1391.586667	1325.362444	4317.228667
28	lyso phosphatidic acids	$C_{18}H_{37}O_7P_1$	2559.235667	716.2901111	1519.219889	1608.574556	3720.801556	1734.248667
29	lyso phosphatidic acids	$C_{19}H_{37}O_7P_1$	7851.901667	2388.148	5201.919222	6745.754667	5855.585111	8123.035222
30	lyso phosphatidic acids	$C_{21}H_{39}O_7P_1$	2055.023667	2135.680222	1698.616222	2176.041667	1358.656	2817.901778

31	lyso phosphatidic acids	C23H45O4P1	4438.842667	479.2681111	1322.647222	2127.071333	1755.549333	2379.719556
32	lyso phosphatidic acids	C27H49O8P1	5420.627667	64.99022222	2537.607556	3025.258111	3052.888222	3424.313333
33	lyso phosphatidylethanolamines	C17H34O7N1P1	2296.246833	380.416	1134.820444	1404.077889	1763.300444	2167.503
34	lyso phosphatidylethanolamines	C18H36O7N1P1	2266.150667	545.007	1273.273222	2144.028667	2202.640889	2625.089778
35	lyso phosphatidylethanolamines	C18H38O7N1P1	3481.065833	241.7228889	1156.566667	1931.287222	2280.051222	2920.651
36	lyso phosphatidylethanolamines	C20H40O7N1P1	25198.84183	11167.395	11430.75611	19245.75844	16252.30856	14814.42756
37	lyso phosphatidylethanolamines	C22H43O7P1	13738.9395	63.23922222	1263.636889	12315.00678	1859.224667	2081.679111
38	lyso phosphatidylethanolamines	C21H44O7N1P1	2974.205167	40.28311111	1730.783222	2148.688556	2420.572556	2407.276222
39	lyso phosphatidylethanolamines	C27H54O7N1P1	13491.29167	129.0242222	4156.409778	7104.868111	6739.756	11572.95144
40	lyso phosphatidylethanolamines	C27H56O7N1P1	16869.19233	264.1431111	4075.438667	3422.928556	9695.410444	5940.840222
41	lyso phosphatidylethanolamines	C33H68O7N1P1	8021.465667	520.1468889	3270.420667	1385.802444	1380.704889	6850.237667
42	lyso phosphatidylethanolamines	C37H76O7N1P1	5907.663333	4761.915222	5438.557778	4620.669111	3979.087222	4927.722111
43	lyso phosphatidylglycerols	C22H43O9P1	3493.645333	545.5215556	1470.586556	2125.260444	2127.064222	2811.282778
44	lyso phosphatidylglycerols	C24H49O9P1	5124.008167	71.03222222	1495.011778	1603.736556	1510.031667	2709.412222
45	lyso phosphatidylglycerols	C26H53O9P1	2133.600333	85.49988889	2215.928889	2320.032889	2466.207444	2496.608667
46	lyso phosphatidylglycerols	C23H43O12P1	9577.946667	593.2425556	2316.124556	832.5598889	884.987	4834.855222
47	lyso phosphatidylglycerols	C28H57O9P1	4162.138333	280.7383333	2062.508667	3716.483111	2242.841111	3408.108889
48	lyso phosphatidylglycerols	C31H60O8N1P1	4092.978333	147.7483333	2006.034	3198.285111	3066.498222	2779.681333
49	lyso phosphatidylglycerols	C32H65O9P1	5098.3815	368.2424444	1394.643556	3049.049444	3482.903556	4782.320889
50	lyso phosphatidylglycerols	C33H63O10P1	18798.26267	2138.775	15462.365	12273.575	11069.41378	3290.454444
51	lyso phosphatidylinositols	C27H53O12P1	5864.3505	1593.867778	5226.668	4878.645556	4049.903889	4266.042556
52	lyso phosphatidylinositols	C29H57O12P1	3588.538833	76.15477778	1467.909111	1624.964111	2488.47	2270.589111
53	lyso phosphatidylinositols	C35H67O12P1	2339.101	422.4462222	1478.264	2792.526444	2010.506333	3265.335111
54	lyso phosphatidylinositols	C39H77O12P1	2299.893167	1901.867222	1435.439333	2043.221	1697.103556	2631.435444
55	lyso phosphatidylinositols	C40H77O12P1	22290.6505	322.242	12934.59	16688.43167	17606.79367	22497.98222

56	lyso phosphatidylinositols	C40H79O12P1	16329.1625	2514.478111	10753.16789	17420.43267	12576.199	21978.43867
57	lyso phosphatidylinositols	C40H75O13P1	2371.855	952.3436667	1888.553444	2383.993222	1447.446444	2591.033556
58	mannosylphosphomycoketides	C42H85O9P1	2540.029333	282.0102222	1105.698111	1269.232333	1075.821889	2556.509222
59	menaquinones	C36H50O2	8763.914	2612.195556	5367.465333	7813.343889	9704.595111	7814.254889
60	monoacylglycerols	C15H30O4	2538.158	57.65777778	1189.682111	1482.051556	1804.633333	2840.445889
61	monoacylglycerols	C17H32O4	2402.844	1482.991222	2472.132444	1426.926778	1590.090556	3619.915333
62	mycobactins	C42H68O9N5	17401.00633	1134.294778	4937.872889	6967.819222	10114.05767	7784.143222
63	mycobactins	C40H75O13P1	2654.128667	351.1908889	1502.204889	2942.864444	3334.051333	3309.999889
64	mycobactins	C44H72O10N5	6121.498	838.4085556	4708.508444	4724.493111	5957.743222	4323.674222
65	mycocerosic acids	C26H52O2	3708.559333	705.1456667	1497.204556	2710.777111	2496.371111	3359.816222
66	mycolactones	C44H66O8	21420.05417	700.4388889	16656.20489	16863.73811	15021.21622	7081.933222
67	mycolactones	C44H6809	17519.62517	2131.675333	3580.375111	10128.67667	2662.624889	11946.706
68	mycolipodienic acid	C27H50O2	14905.70917	4459.097111	5221.485222	16049.86233	2547.726889	2666.635556
69	phophomycoketides	C31H65O4P1	326412.2063	3191.631556	259902.7829	1027567.533	214155.869	580704.1083
70	phosphatidic acids	C30H55O8P1	2033.161	25.54877778	1650.800667	1620.048	2314.905667	2259.736778
71	phosphatidic acids	C31H61O8P1	1375.417333	548.6526667	937.8443333	1462.332444	1429.824667	1556.879889
72	phosphatidic acids	C32H59O8P1	1545.226667	833.7803333	1634.019222	1544.413778	1141.214667	1857.209444
73	phosphatidic acids	C33H61O8P1	3244.751	850.9115556	1759.312222	2257.314333	2680.885556	4818.946889
74	phosphatidic acids	C33H63O8P1	7055.546833	475.6962222	4143.770444	4831.416667	5289.733444	3520.186333
75	phosphatidic acids	C34H67O8P1	2257.840167	1116.610889	693.8508889	2011.602667	2163.789778	2507.514222
76	phosphatidic acids	C43H69O8P1	8011.9635	322.7014444	3542.528	6448.568111	5427.703333	7840.167556
77	phosphatidylethanolamines	C32H62O8N1P1	34600.23883	3803.337	8196.558778	21045.52489	22036.90111	31677.348
78	phosphatidylethanolamines	C34H68O8N1P1	2805.402167	435.1222222	1437.129444	1723.176444	1854.310889	2256.568222
79	phosphatidylethanolamines	C36H70O8N1P1	2595.061167	541.3342222	1432.504111	1767.303333	2138.139889	3779.614111
80	phosphatidylethanolamines	C39H74O8N1P1	1579.534833	913.959	966.7985556	1152.424667	1702.649889	1652.889333
81	phosphatidylethanolamines	C41H82O8N1P1	2194.264667	2600.583	1245.787889	2953.145444	2274.801667	2357.215111
82	phosphatidylglycerols	C31H59O10P1	3393.892667	965.412	961.2743333	2813.921778	2478.772889	3780.025444
83	phosphatidylglycerols	C34H67O9P1	5195.1015	907.694	2282.226444	5383.772	5183.381	4807.577556
84	phosphatidylglycerols	C34H67O10P1	5141.786	55.316	2918.31	5652.178	4042.171	3811.906444
85	phosphatidylglycerols	C36H69O10P1	16142.24883	472.0748889	11006.12556	10172.99333	12771.921	19235.20567
86	phosphatidylinositols	C35H63O13P1	3746.742667	322.2691111	1590.728	1906.929778	2945.642222	15858.22033
87	phosphatidylinositols	C40H75O13P1	11855.05967	93.71922222	6796.921444	9812.458333	9367.832222	10364.55922

88	phosphatidylinositols	$C_{14}H_{77}O_{13}P_1$	10870.6695	1103.737333	3430.001889	6033.206889	9207.498556	4741.863778
89	triacylglycerols	$C_{39}H_{70}O_6$	1892.1415	1535.617556	3550.733111	1999.002111	1686.992222	2098.061556
90	triacylglycerols	$C_{39}H_{70}O_6$	2301.8105	1532.196333	1470.680556	2081.995889	2643.695333	1995.839778
91	triacylglycerols	$C_{40}H_{76}O_6$	3782.260833	499.6344444	978.3556667	3861.186556	2093.984111	3934.552222
92	triacylglycerols	$C_{45}H_{86}O_6$	2478.333667	78.47633333	1182.514889	1867.744889	2285.383889	3351.648556
93	triacyltrehaloses	$C_{67}H_{124}O_6$	162267.6663	39713.26233	59578.70911	29212.56389	40325.76378	45315.39378
94	triacyltrehaloses	$C_{63}H_{112}O_{14}$	2456.236833	2132.441667	2105.282667	2912.506889	3429.075556	2294.253667

Supplementary Table 1: Intensities of the non-mycolic acid lipids across various conditions identified. Peak intensity across different conditions are depicted from column 4 – 9.

Sl. no.	Elemental composition	Normoxia (Control)	Dormancy	R24	R48	R72	R144
1	$C_{53}H_{102}O_5$	2959.146778	174.5805556	4814.061	2457.936889	3623.987778	2901.434667
2	$C_{56}H_{110}O_3$	4840.279333	825.3247778	363.3216667	1201.594556	6192.245111	2922.021333
3	$C_{54}H_{104}O_5$	2022.593222	380.1637778	896.9921111	2433.739815	1819.41	1514.579
4	$C_{56}H_{106}O_5$	4385.622778	988.6336667	1087.140444	1275.565259	3866.279556	3142.363778
5	$C_{58}H_{114}O_3$	11592.19211	981.5908889	3240.025556	5092.362778	9289.412778	1551.678444
6	$C_{56}H_{108}O_5$	37796.696	2720.247111	45174.89178	18357.75781	13619.63589	2424.744222
7	$C_{60}H_{118}O_3$	2570.176667	126.378	1537.738889	3791.488333	3296.972667	1960.343222
8	$C_{58}H_{112}O_5$	5370.906333	216.4327778	1226.955556	2940.54063	3739.396222	4726.093111
9	$C_{59}H_{114}O_5$	1987.062	78.29833333	215.4721111	776.329037	1562.983889	2480.743778
10	$C_{62}H_{112}O_3$	1703.549	193.9026667	1343.369778	1970.45963	1898.890778	1192.476111
11	$C_{60}H_{116}O_5$	2823.118111	152.2597778	1113.501667	1443.826407	2947.477667	3345.638222
12	$C_{61}H_{118}O_5$	3485.599556	34.93411111	1393.648556	1260.022704	2254.066556	2703.723444
13	$C_{64}H_{126}O_3$	2642.355444	239.0002222	983.6192222	2400.027	1892.647111	3065.833444
14	$C_{62}H_{120}O_5$	3695.965222	571.9181111	1322.291111	1504.264889	1915.004778	2530.977
15	$C_{63}H_{122}O_5$	2362.821667	487.3728889	673.3253333	554.881037	1734.295444	2865.721889

16	C68H134O3	4664.350556	124.2974444	343.193	616.9785926	4906.564556	3663.333444
17	C70H136O3	1296.780778	1265.234889	1438.777889	2292.687481	2889.102111	1448.538667
18	C69H134O5	2914.723444	291.1363333	730.7022222	363.4857778	2750.459111	2099.792222
19	C71H146O4	25776.29356	32078.776	26091.85256	9260.917963	22760.34744	32996.07633
20	C73H142O3	4486.264778	377.9052222	1240.315222	2321.226889	3082.991	2800.712
21	C72H140O4	793.8015556	1395.964111	1036.198556	1514.501889	1961.477222	1452.748667
22	C74H144O3	11131.24922	3065.201778	1541.873222	2728.597407	2146.911444	7232.566111
23	C73H142O4	419351.4404	347284.9498	361729.49	314785.0884	377465.51	353297.7634
24	C73H142O4	419351.4404	347284.9498	361729.49	314785.0884	377465.51	353297.7634
25	C73H144O4	39212.32644	17528.36244	11337.105	10428.98219	16723.542	5984.739444
26	C73H148O4	5878.612889	481.6664444	447.1773333	516.331037	3578.333667	1838.018889
27	C73H150O4	4434.761222	953.2551111	1631.753111	2085.528593	3544.798222	3933.655889
28	C74H144O4	6572.071778	749.7028889	2071.132111	2658.832407	4919.987333	7661.806
29	C74H144O4	6572.071778	749.7028889	2071.132111	2658.832407	4919.987333	7661.806
30	C74H144O4	6572.071778	749.7028889	2071.132111	2658.832407	4919.987333	7661.806
31	C74H146O4	10817.52811	4487.269222	2624.300444	2501.621556	6271.668222	13130.27356
32	C75H152O4	5247.903111	51698.158	17200.42744	4561.202148	3417.363556	3485.176444

33	C78H152O3	7434.587444	2775.969444	3138.894778	7183.041444	1988.589333	4709.871333
34	C77H152O3	529.6085556	7291.442	3257.812667	691.3299259	1808.306222	1801.016111
35	C77H152O3	529.6085556	7291.442	3257.812667	691.3299259	1808.306222	1801.016111
36	C77H152O3	529.6085556	7291.442	3257.812667	691.3299259	1808.306222	1801.016111
37	C77H152O4	3646.188556	605.6167778	748.3623333	3607.33437	2533.173556	3793.820778
38	C77H152O4	3646.188556	605.6167778	748.3623333	3607.33437	2533.173556	3793.820778
39	C78H152O4	2470.265444	279.751	1080.597667	1325.733519	1477.306889	2017.060222
40	C78H152O4	2470.265444	279.751	1080.597667	1325.733519	1477.306889	2017.060222
41	C81H158O3	1494.634333	894.4004444	3339.889889	2224.896741	1827.611222	1964.062556
42	C80H156O4	14068.61956	537.014	2849.195556	5131.345889	9686.508	11283.13211
43	C80H156O4	14068.61956	537.014	2849.195556	5131.345889	9686.508	11283.13211
44	C80H156O4	14068.61956	537.014	2849.195556	5131.345889	9686.508	11283.13211
45	C80H158O4	1429.819111	4557.260778	363.6585556	1308.167222	4289.894444	1920.377111
46	C82H160O3	3955.789333	412.3308889	1780.114667	2534.126407	2521.963444	5760.707111
47	C83H162O3	5396.788889	411.2201111	525.815	2292.166148	3739.119	2419.227778
48	C83H158O4	3071.906	720.8685556	1877.104444	788.9514444	2940.074	3270.701
49	C83H162O4	828.192	1324.242556	661.6643333	449.7757407	1336.673889	2034.019556

50	C83H162O4	828.192	1324.242556	661.6643333	449.7757407	1336.673889	2034.019556
51	C83H162O4	828.192	1324.242556	661.6643333	449.7757407	1336.673889	2034.019556
52	C83H64O4	7161.705667	400.5178889	1447.342778	4656.924444	3275.885222	5717.076111
53	C84H164O4	5025.370667	198.4546667	401.769	2651.695778	3968.061889	16409.43556
54	C84H164O4	5025.370667	198.4546667	401.769	2651.695778	3968.061889	16409.43556
55	C85H160O4	248.2414444	1534.007222	1860.589889	3754.247556	1567.632667	1005.775889
56	C85H168O4	6661.376667	69093.34311	4266.157889	5568.759111	4389.960556	2177.582444
57	C87H170O3	5117.750111	592.2041111	2157.548889	5427.435444	3411.473111	3898.599
58	C87H162O4	2175.988889	1279.365556	1384.271444	1405.434037	1876.370222	2576.564333
59	C88H172O3	1654.312333	275.605	1461.100333	1460.128481	1617.465222	1699.024222
60	C87H170O4	2733.275333	735.1845556	5175.261222	1773.977111	1480.750111	1644.404222
61	C87H170O4	2733.275333	735.1845556	5175.261222	1773.977111	1480.750111	1644.404222
62	C87H170O4	2733.275333	735.1845556	5175.261222	1773.977111	1480.750111	1644.404222
63	C87H172O2	2797.710667	1683.760444	2044.665778	1977.595185	1855.642667	1589.521
64	C88H164O4	14571.47533	18439.48011	10147.96756	4480.546407	7839.292	27633.714
65	C89H174O3	1889.275333	93.46511111	3367.541	4321.863481	1784.801444	6978.470444
66	C88H172O4	2358.938444	414.5651111	593.7194444	1588.658963	1719.013111	2257.423556

67	C88H172O4	2358.938444	414.5651111	593.7194444	1588.658963	1719.013111	2257.423556
68	C88H174O4	14155.95178	300.7283333	2938.091889	2608.535259	4201.797444	39354.218
69	C89H166O4	11370.20656	1293.060333	1298.079667	1796.339889	4783.721444	15604.18533
70	C91H180O4	1142.374667	1369.994111	3377.76	1800.918481	1585.513333	1460.731444

Supplementary Table 2: Intensities of the mycolic acid across various conditions identified. Peak intensity across different conditions are depicted from column 3 – 8.
